# Supplementary material for: Uncovering the chemistry of C–C bond formation in C-nucleoside biosynthesis: crystal structure of a C-glycoside synthase/PRPP complex
Source: Chem Commun (Camb). 2020 Jun 9;56(55):7617–20. doi: 10.1039/d0cc02834g (PMC8183095; doi:10.1039/d0cc02834g)
Supplement: CC-056-D0CC02834G-s001 [file CC-056-D0CC02834G-s001.pdf]

Electronic Supplementary Material (ESI) for ChemComm.
This journal is © The Royal Society of Chemistry 2020

Uncovering the Chemistry of C-C Bond Formation in C-Nucleoside Biosynthesis: Crystal Structure of a *C*-Glycoside Synthase/PRPP Complex

Sisi Gao,[a,b] Ashish Radadiya,[c] Wenbo Li,[d] Huanting Liu,[b] Wen Zhu,[e] Valérie de Crécy-Lagard,[f] Nigel G. J. Richards*[c,g] and James H. Naismith*[d,h]

[a] Research Complex at Harwell, Didcot, OX11 0FA, UK

[b] BSRC, University of St. Andrews, St. Andrews, KY16 9ST, UK

[c] School of Chemistry, Cardiff University, Cardiff, CF10 3AT, UK

[d] Division of Structural Biology, University of Oxford, Oxford, OX3 7BN, UK

[e] Department of Chemistry and California Institute for Quantitative Biosciences, University of California, Berkeley, CA 94720, USA

[f] Department of Microbiology, University of Florida, Gainesville, FL 32611, USA

[g] Foundation for Applied Molecular Evolution, Alachua, FL 32415, USA

[h] The Rosalind Franklin Institute, Didcot, OX11 0FA, UK

Supporting Information

Full citations for references 2 and 3 in the main text:

T. K. Warren, R. Jordan, M. K. Lo, A. S. Ray, R. L. Mackman, V. Soloveva, D. Siegel, M. Perron, R. Bannister, H. C. Hui, N. Larson, R. Strickley, J. Wells, K. S. Stuthman, S. A. Van Tongeren, N. L. Garza, G. Donnelly, A. C. Shurtleff, C. J. Retterer, D. Gharaibeh, R. Zamani, T. Kenny, B. P. Eaton, E. Grimes, L. S. Welch, L. Gomba, C. L. Wilhelmsen, D. K. Nichols, J. E. Nuss, E. R. Nagle, J. R. Kugelman, G. Palacios, E. Doerffler, S. Neville, E. Carra, M. O. Clarke, L.-J. Zhang, W. Lew, B. Ross, Q. Wang, K. Chun, L. Wolfe, D. Babusis, Y. Park, K. M. Stray, I. Trancheva, J. Y. Feng, O. Barauakas, Y.-L. Xu, P. Wong, M. R. Braun, M. Flint, L. K. McMullan, S. S. Chen, R. Fearns, S. Swaminathan, D. L. Mayers, C. F. Spiropoulou, W. A. Lee, S. T. Nichol, T. Cihlar and S. Bavari, *Nature*, 2016, **531**, 381.

M. L. Agostini, E. L. Andres, A. C. Sims, R. L. Graham, T. P. Sheahan, X.-T. Lu, E. C. Smith, J. B. Case, J. Y. Feng, R. Jordan, A. S. Ray, T. Cihlar, D. Siegel, R. L. Mackman, M. O. Clarke, R. S. Baric and M. R. Denison, *MBio*, 2018, **9**, e00222-18.

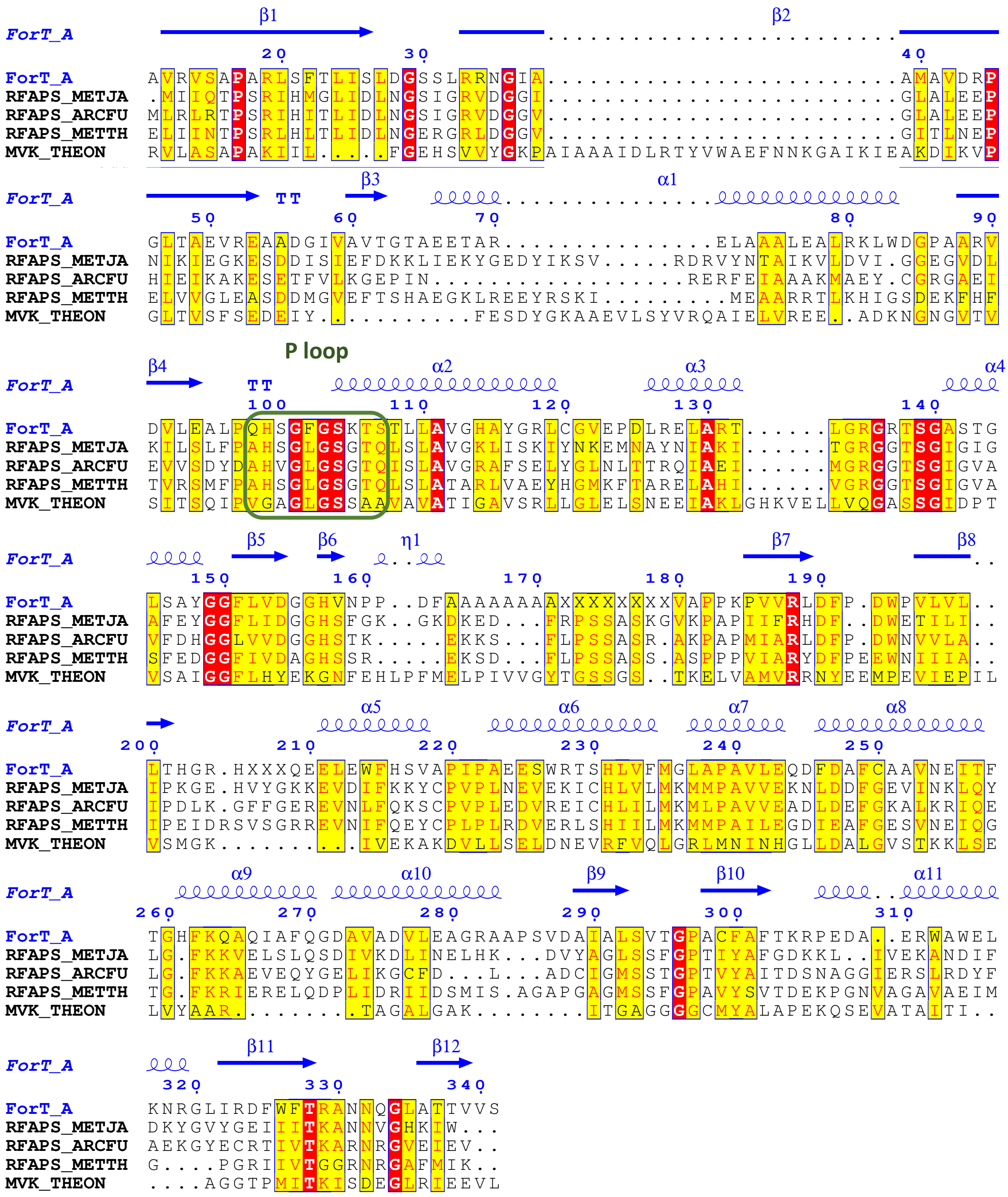

Figure S1 Protein sequence alignment of ForT with other members of the GHMP kinase superfamily.[1] RFAPS_METJA, RFAPS_ARCFU and RFAPS_METTH are homologues of RFA synthases from different organisms (*Methanocaldococcus jannaschii*,[2] *Archaeoglobus fulgidus* and *Methanothermobacter thermautotrophicus*, respectively). MVK_THEON (*Thermococcus onnurineus*) is a mevalonate kinase.[3] Conserved residues are shown in yellow boxes with identical shaded in red. The secondary structure of ForT is labelled above the sequence alignment in blue. (pyro)phosphate-binding motif is outlined by the green box. Figure was generated using Endscript 2.0.[4]

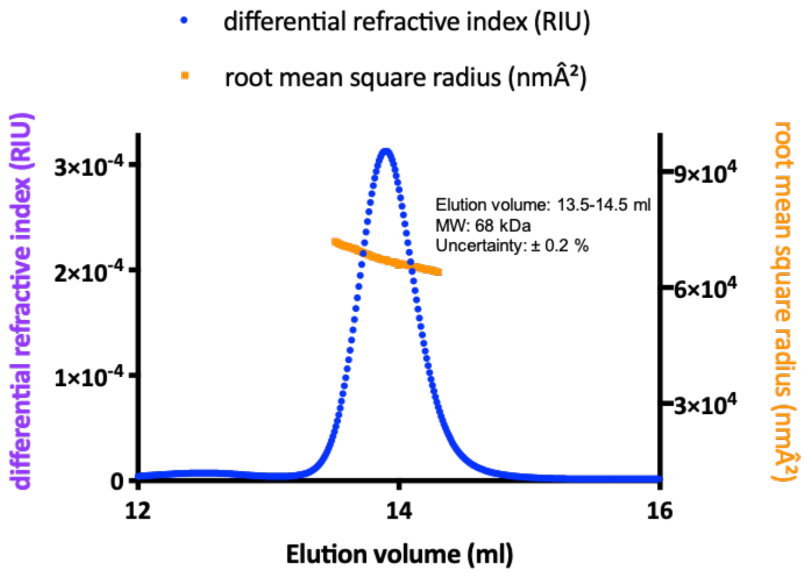

Figure S2 SEC-MALS chromatography of recombinant, WT ForT. The SEC-MALS spectrum demonstrates that ForT is eluted as a homogeneous species. Changes in the refractive index (blue) show protein-mediated scattering of light. The root-mean-square radius (orange) indicates the molecular size of the enzyme in solution, which is measured to be 68 kDa (0.2 % uncertainty). These findings support the hypothesis that ForT (36 kDa) is a dimer in solution. ForT-PRPP complex was subjected to the SEC-MALS in the same manner and a similar result was observed. We take these data to be strong experimental evidence that the protein exists as a dimer in solution.

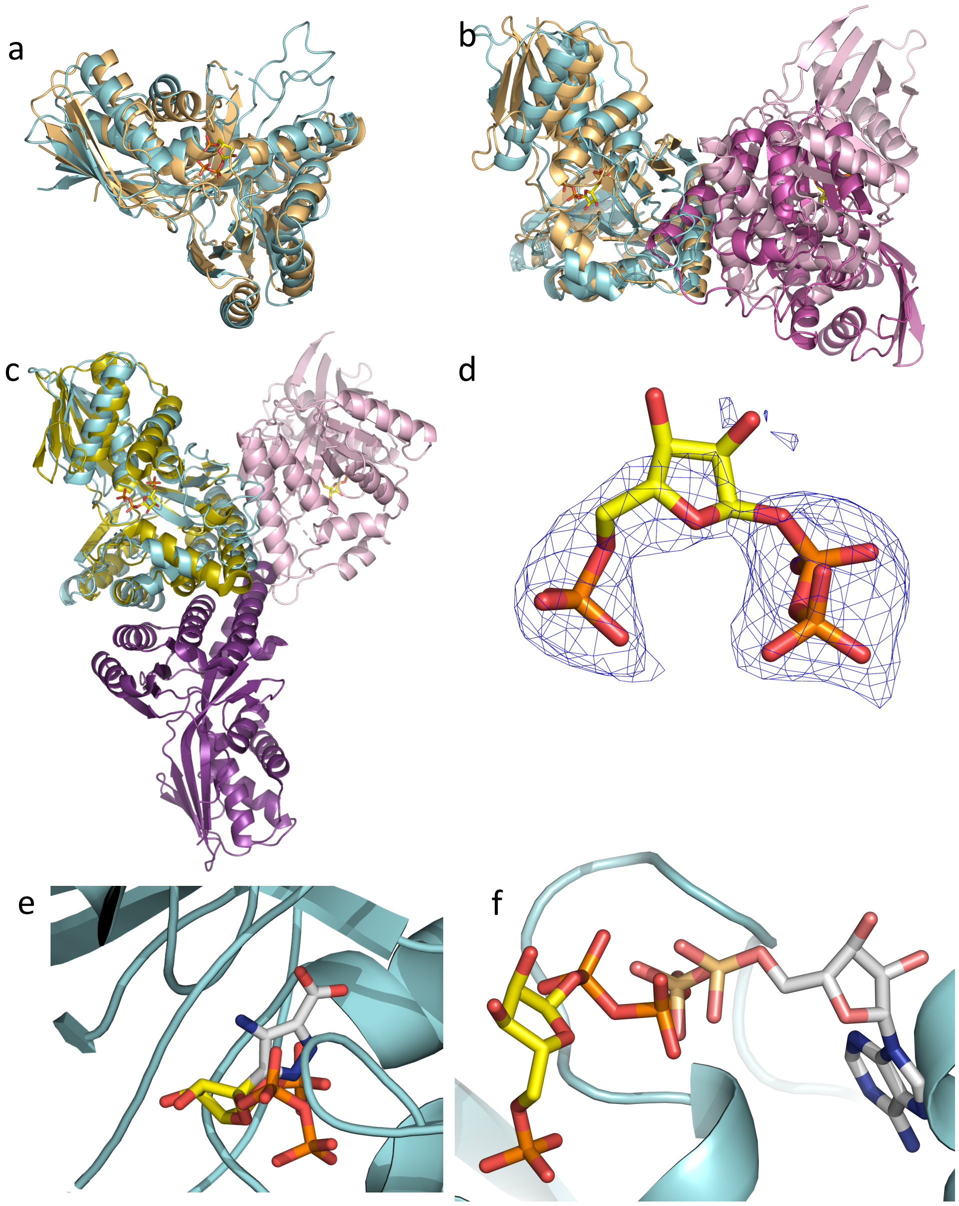

Figure S3 Additional structural data. (a) The ForT monomer, (colored cyan) shares a fold with homoserine kinase (PDB 1FWL, colored wheat).[1] (b) Homoserine kinase uses the helical bundle to mediate contacts between monomers in its dimer (wheat and magenta) in a similar manner to ForT. The dimeric arrangement of the homoserine kinase, however, shows an approximately 60° rotation from that in ForT (cyan and pale pink). (c) Mevalonate kinase (PDB 6MDE, olive and dark magenta)[5] shares the same monomeric structure as ForT but has a very different dimeric arrangement. (d) The original (unbiased) Fo-Fc map contoured at 3 σ showing clear density for the phosphate and pyrophosphate. These were included in the model for refinement. The density was weaker for ribose and this was not included in the model until later cycles of refinement. (e) The hybrid computational probe **7** (main text) clashes with a loop in the protein suggesting that conformational changes take place during as a result of substrate binding and during catalysis. (f) Overlap of the homoserine kinase ADP complex (PDB 1FWK)[1] and ForT shows that the β-phosphate of ADP overlaps with the distal phosphate of the pyrophosphate of PRPP. Carbon atoms in ADP are colored grey; other atoms are colored as shown in Figure 4a (main text).

(a) ITC measurements

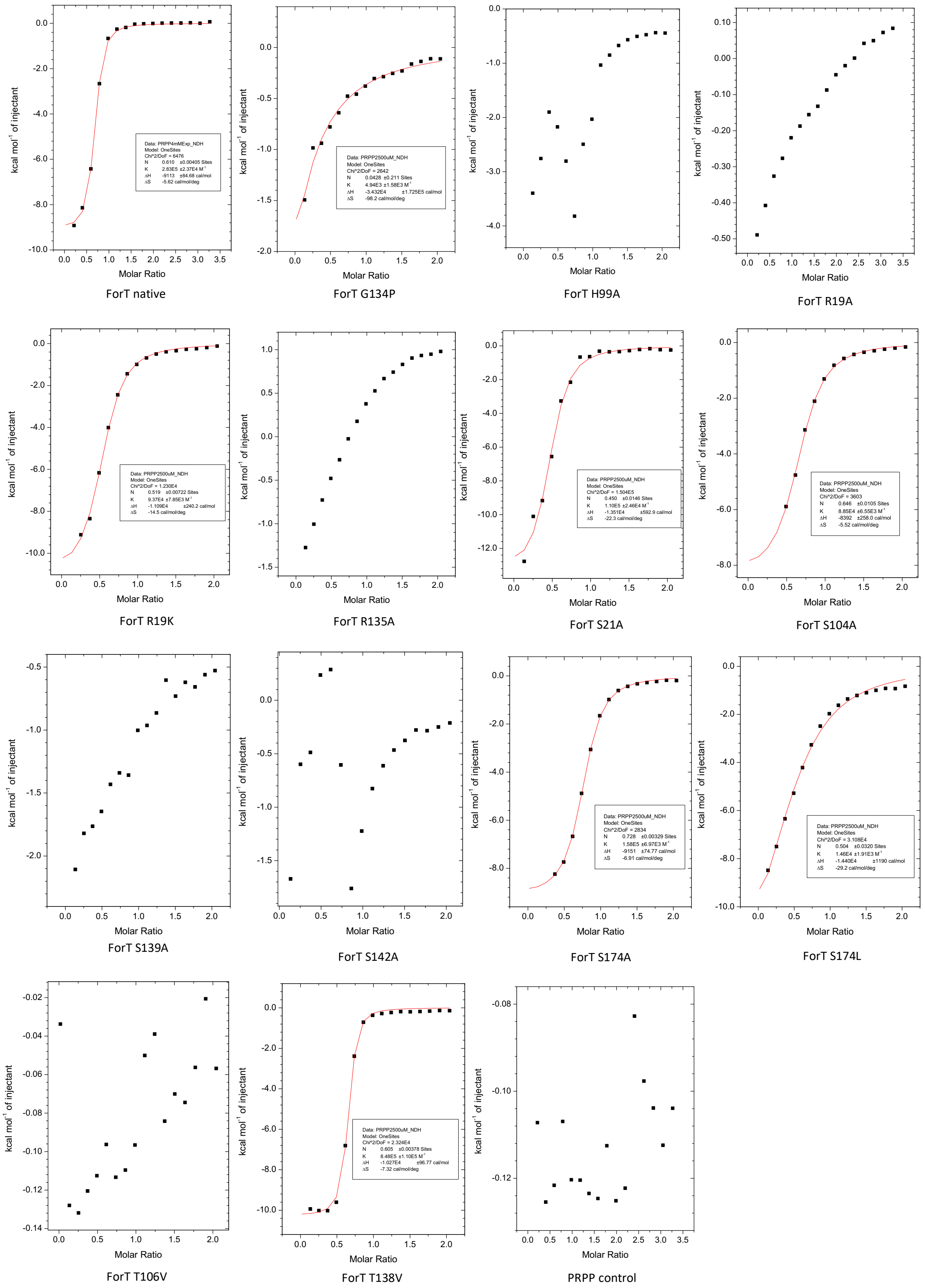

(b) LCMS-based enzyme assay

Mass detection set for 340 (MSD2)

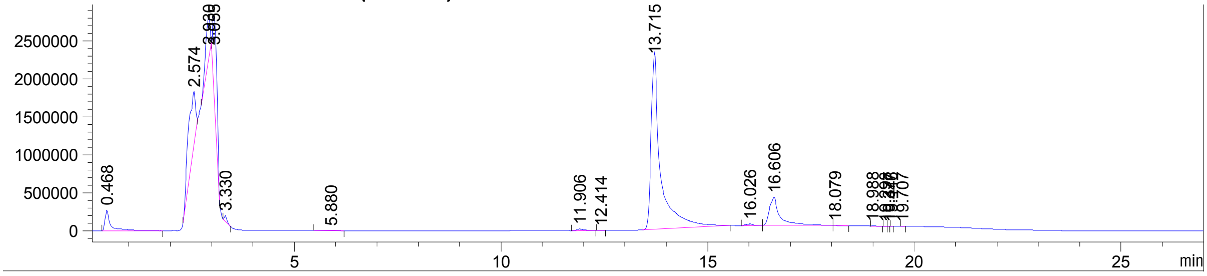

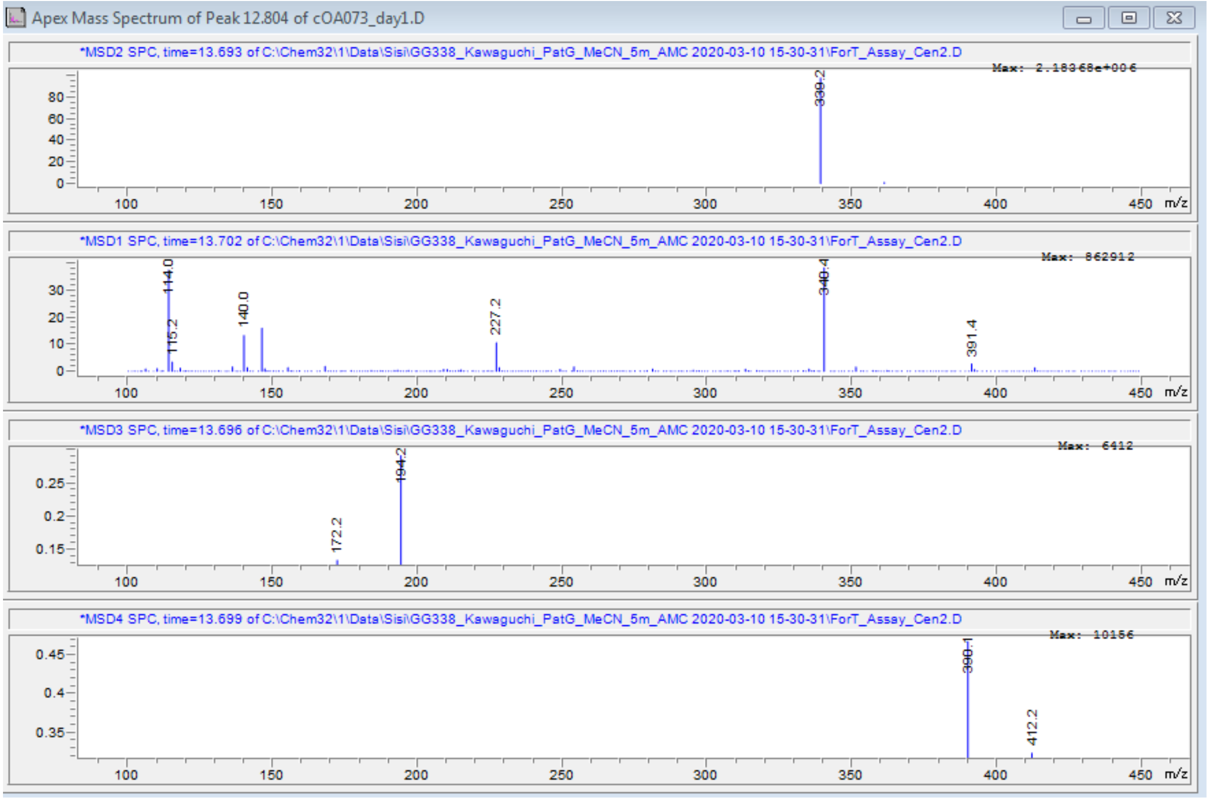

MSD1: Total Mass scan; MSD2: Mass detection for product **6**; MSD3: Mass detection for APDA **5**; MSD4: Mass detection for PRPP **4**.

(c) MIMS-based enzyme assay of WT ForT activity

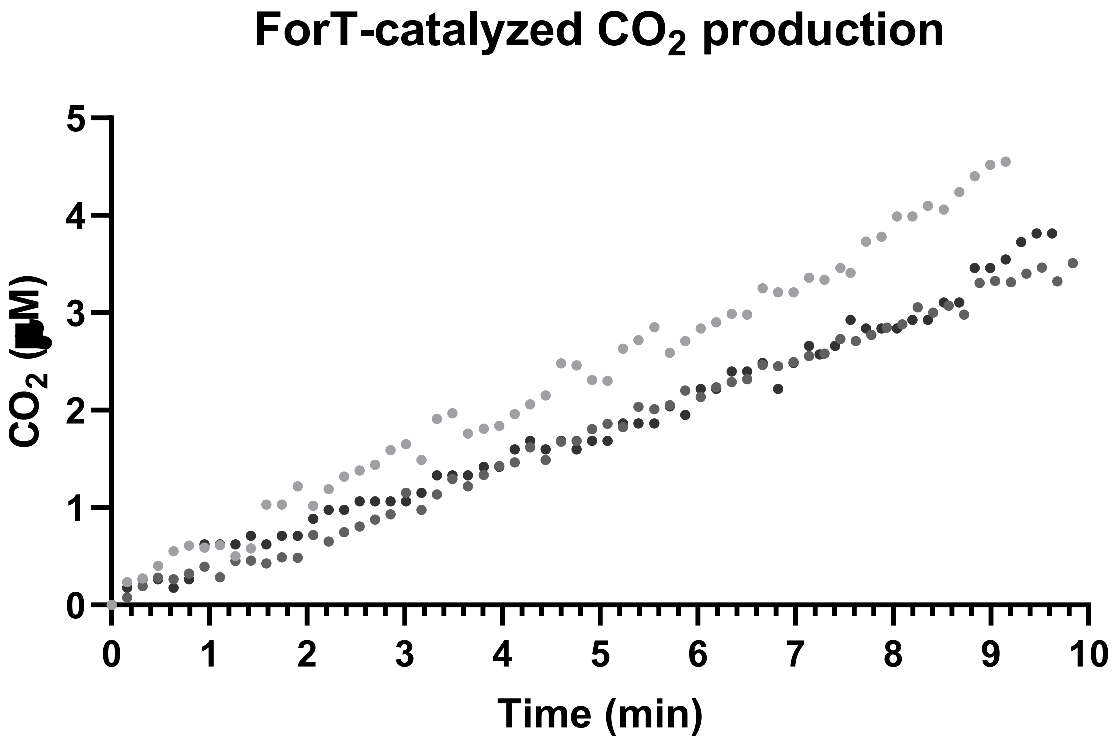

Figure S4 Biochemical characterisation of the enzyme. (a) Raw ITC data used to obtain the K_D values for PRPP binding (Table S2). (b) LCMS data showing that incubation of ForT with PRPP **4** and ADPA **5** gives the expected product **6** (Figure 1, main text). (c) MIMS data (triplicate runs) used to obtain the specific activity of WT ForT (Table S2).

Table S1 Data collection and refinement statistics.

	ForT-PRPP
Beamline	i04-1 (DLS)
Wavelength (Å)	0.9159
Space group	$P4_12_12$
a, b, c (Å)	81.1, 81.1, 111.0
α, β, γ (°)	90, 90, 90
Resolution range (Å)	65.5 – 2.5 (2.57 – 2.50)
R_{meas}	0.066 (2.15)
I/ σ (I)	13.2 (1.1)
Completeness (%)	100 (99)
Multiplicity	12.5 (13.1)
No. reflections	287431 (14481)
R_{work}	0.227 (0.225)
R_{free}	0.27 (0.42)
Number of atoms	
Total	2435
Protein	2401
Ligands / ions	28
Waters	6
B-factor	
Average	62
Protein only	62
Ligands/Ion	97
Water	72.1
Bond lengths (Å)	0.005
Bond angles (°)	1.13
RSCC of PRPP (occupancy 1)	0.893
Ramachandran plot favoured / outlier (%)	94.3 / 2.6

Table S2 Binding affinity of PRPP **4** (determined by ITC) and specific activities (determined by MIMS assay) for WT ForT and site-specific ForT variants. Data for experiments involving WT ForT are shown in Figure S4.

Enzyme	K_D (μM)	Activity (U/mg)
WT ForT	3.6 ± 0.3	0.002
T138V	1.2 ± 0.2	Not determined
S174A	6.34 ± 0.3	0.0003
S21A	9.6 ± 2.1	Unstable under the assay conditions
R19K	10.8 ± 0.9	Unstable under the assay conditions
S104A	11.4 ± 0.8	0.0007
S174L	70 ± 9	Unstable under the assay conditions
S139A	> 100	Unstable under the assay conditions
G134P	> 200	Not determined
H99A	No binding	Unstable under the assay conditions
R19A	No binding	No activity
R135A	No binding	Unstable under the assay conditions
S142A	No binding	Not determined
T106V	No binding	Not determined

Table S3 Primers used for the site-directed mutagenesis of ForT.

Primers	Sequences (5'-3')
R19A-f	TGCTCCAGCAGCGCTTAGCTTCACGCTGATTAGCCTTGAC
R19A-r	GCTAAGCGCTGCTGGAGCACTGACCCGAACCGCGC
R19K-f	TGCTCCAGCAAAACTTAGCTTCACGCTGATTAGCCTTGACGG
R19K-r	GCTAAGTTTTGCTGGAGCACTGACCCGAACCGCGCCG
S21A-f	CAGCAAGACTTGCGTTCACGCTGATTAGCCTTGACGGCAGTTCC
S21A-r	GCGTGAACGCAAGTCTTGCTGGAGCACTGACCCGAACCGC
H99A-f	CCTTCCCCAAGCGAGCGGTTTTGGATCTAAAACTAGTACCTTGTTG
H99A-r	CCGCTCGCTTGGGGAAGGGCCTCAAGTACGTCGACACG
S104A-f	GGTTTTGGAGCGAAAACTAGTACCTTGTTGGCTGTAGGACATGCC
S104A-r	CTAGTTTTCGCTCCAAAACCGCTGTGTTGGGGAAGGGCCTCAA
T106V-f	GGATCTAAAGTGAGTACCTTGTTGGCTGTAGGACATGCCTAC
T106V-r	CAAGGTACTCACTTTAGATCCAAAACCGCTGTGTTGGGGAAG
G134P-f	CGTACCTTACCGCGGGGTCGTACCTCAGGTGCTAGTACAG
G134P-r	CCCCGCGGTAAGGTACGAGCAAGCTCGCGCAGGTCAGG
R135A-f	CCTTAGGTGCGGGTCGTACCTCAGGTGCTAGTACAGGC
R135A-r	TACGACCCGCACCTAAGGTACGAGCAAGCTCGCGCAG
T138V-f	GGTCGGGGTCGTGTGTCAGGTGCTAGTACAGGCCTTTCCGCCTATG
T138V-r	AGCACCTGACACACGACCCCGACCTAAGGTACGAGCAAGCTC
S139A-f	GTCGTACCGCGGGTGCTAGTACAGGCCTTTCCGCCTATGG
S139A-r	CTAGCACCCGCGGTACGACCCCGACCTAAGGTACGAGCAAG
S142A-f	AGGTGCTGCGACAGGCCTTTCCGCCTATGGCGGATTTCTG
S142A-r	AAAGGCCTGTCGCAGCACCTGAGGTACGACCCCGACCTAA
S174A-f	CGCCCGGCGCGTTTCGCACAGCAAGTAGCTCCGCCTAAG
S174A-r	GCGAAACGCGCCGGGCGTAAATACTTTTGTGGAGCCTCTGCG
S174L-f	CGCCCGCTGCGTTTCGCACAGCAAGTAGCTCCGCCTAAG

General methods.

Unless specified, all chemicals were purchased from Sigma-Aldrich or Fisher Scientific. Oligonucleotides were purchased from Integrated DNA Technologies. dNTP and DNA polymerase were purchased as part of the EMD Millipore Novagen KOD Hot Start DNA Polymerase kit. DNA sequencing was performed by Source Bioscience.

Gene cloning and PCR.

ForT was cloned into pEHISTEV vector with a N terminal His tag followed by a TEV protease cleavage site.[6]

Site-directed mutagenesis.

Mutations of ForT were cloned using an established site-directed mutagenesis protocol.[7] Primer pairs (around 30 nucleotides) carrying the overlapping mutation (Table S3) and Q5 DNA polymerase (New England Biolabs) were employed for amplification (94 °C, 7 min for initial denaturation, 12 x 95 °C, 1 min denaturation followed by T_m - 5 °C 1 min annealing and 68/72 °C 5.5 min extension, 68/72 °C 10 min final extension). The PCR product was DpnI digested for 1 hour at 37 °C and 5 µl of the digested product was used for transformation of *Escherichia coli* DH5α. All mutations were verified by DNA sequencing.

Protein expression.

ForT and its variants were transformed into *E. coli* BL21 (pLysS) competent cells and plated onto LB agar containing 50 µg/ml kanamycin and 25 ng/ml chloramphenicol. Transformation plate was incubated at 37 °C overnight. A single colony was picked and inoculated into 100 ml LB medium supplemented with the same concentration of antibiotics, after growing overnight in an incubator at 37 °C with a shaking speed of 200 rpm, 10 ml culture was inoculated into 1 L LB medium containing the same concentration of antibiotics. Cultures were incubated under 37 °C, 200 rpm, until the optical density OD_{600} reached 0.6, then 0.4 mM IPTG was added and the cells were further incubated at 20 °C for 20 hours at 200 rpm. The induced cells were harvested by centrifugation and cell pellets were kept at -80°C for protein purification. All ForT variants were expressed using the same procedure.

Protein purification.

Escherichia coli cell pellets over-expressing ForT variants were resuspended in ForT lysis buffer [500 mM NaCl, 50 mM sodium phosphate (pH 7.5), 20 mM imidazole, and 3 mM BME], with addition of Roche EDTA-free protease inhibitor tablets (1 tablet per 50 ml lysis buffer) and DNase at 0.4 mg/g of wet cell pellet. The re-suspension was lysed by being passed through a cell disruptor at 30K psi (Constant Systems). The lysate was cleared by centrifugation at 17,000 rpm, 4 °C for 20 min and then loaded onto a Ni Sepharose 6 FF column (GE Healthcare) equilibrated with lysis buffer. After washing the column with three bed-volumes of 500 mM NaCl, 50 mM sodium phosphate (pH 7.5), 30 mM imidazole, and 3 mM BME, the protein was eluted with 500 mM NaCl, 50 mM sodium phosphate (pH 7.5), 250 mM imidazole, and 3 mM BME and passed through a desalting column (16/10 Desalting, GE Healthcare) with a desalting buffer [100 mM NaCl, 50 mM NaPi (pH 7.5), 3 mM BME], ForT was collected and TEV protease was added at a mass ratio of 10:1. The protein was digested for 3 h at 20 °C before being loaded onto a second nickel column pre-equilibrated with desalting buffer. The second nickel flow-through was then concentrated to 7.5 mL (Vivaspin concentrators, 30 kDa molecular weight cutoff) and applied to a Superdex 200 gel filtration column (GE Healthcare) equilibrated with gel filtration buffer [150 mM NaCl, 10 mM HEPES (pH 7.4), and 1 mM TCEP]. The purity of ForT was analysed by SDS gel electrophoresis and its identity and integrity were further confirmed by mass spectrometry.

Structure Biology

ForT crystallisation was first screened using purified ForT (43mg/ml) premixed with 10 mM PRPP. The best screening hit was obtained in JCSG screen containing 40 mM KH_2PO_4, 20 % (v/v) glycerol, 16 % (w/v) PEG 8000 with 43 mg/ml protein at 20 °C. The hit conditions were further optimised by adding 20 mM Tris-HCl (pH 7.5-9), 20 mM HEPES (pH 7-8), 20 mM Bis-Tris (pH 6-7) or 20 mM Bis-Tris propane (pH 6-8). Bis-tris propane gave the best crystals and we did not see any variation in quality between pH 6 and 8.

ForT crystals were further optimised and finally grown in the hanging drop plates (EasyXtal 15-well DG-Tool X-Seal) using 1 µl of the protein solution (43 mg/ml) mixed with 1 µl of reservoir solution in the hanging drop. The crystals were mounted in a cryo-loop (Molecular Dimensions) and cryo-protected in solutions containing mother liquor plus 200 mM PRPP,

immediately frozen in liquid nitrogen and stored in a cryogenic Dewar. X-ray data were collected at Diamond Light Source I04-1.

The original hit was optimised in the hanging drop plates (EasyXtal 15-well DG-Tool X-Seal) using 1 µl of the protein solution (43 mg/ml) and 1-2 µl of well solution in the hanging drop. The crystals were mounted in a cryo-loop (Molecular Dimensions) and cryo-protected in solutions containing mother liquor plus 200 mM PRPP. The crystals were then frozen by plunging them into liquid nitrogen and sent in a cryogenic Dewar to Diamond Light Source I04-1 for data collection.

The data for the ForT-PRPP complex were collected at Diamond Light Source I04-1 and processed using Diamond online processing software XIA2[8] and DIALS.[9] The complex structure was solved using molecular replacement in PHASER.[10] The model was an unpublished homologue, solved by selenomethionine, which will be published in due course. Ligands were introduced into density during refinement when the F_o-F_c map was judged to be unambiguous in COOT.[11] Crystals grown without PRPP diffracted poorly, electron density maps showed unmodelled density at the active site, thus we have as yet been unable to obtain a true apo structure.

Oligomeric status of ForT.

ForT was subjected to the size-exclusion chromatography multiangle ligand scattering (SEC-MALS)[12] for determination of molecular mass. Purified protein was loaded onto a GE Health Superdex 200 Increase 10/300 GL column, equilibrated in gel filtration buffer (150 mM NaCl, 10 mM HEPES, 1 mM TCEP), attached to the Wyatt Dawn Heleos II Multi-Angel Light Scattering detector and Wyatt Optilab T-rex Refractive Index detector. Protein elution peak was recorded by differential refractive index (dRI).

Isothermal titration calorimetry.

Equilibrium binding experiments with ForT variants and PRPP were performed using a MicroCal PEAQ-ITC (Malvern) in gel filtration buffer (150 mM NaCl, 10 mM HEPES pH 7.5, 1 mM TCEP). All protein samples were buffer-exchanged or dialysed in gel filtration buffer and the proteins were further diluted in the same batch of gel filtration buffer to minimise buffer

mismatches. ITC binding experiments were performed using a cell solution containing 250 μM ForT variants and a titrant solution with 2.5 mM PRPP. The measurements were performed at a stirring speed of 750 rpm at 25 °C using a reference power of 5 μcal/s. A buffer titration control in which compounds were titrated into buffer was performed for each experiment. Data processing, fitting to a nonlinear one-site binding model, and data plotting were performed using MicroCal PEAQ ITC analysis software (Malvern).

LC-MS assay of WT ForT activity.

Based on a literature procedure,[13] 4-amino-3,5-pyrazole dicarboxylic acid (APDA) **5** (2.5 mM) and PRPP **4** (5 mM) were incubated with recombinant, WT ForT (25 μM) in 50 mM sodium phosphate buffer, pH 7.5, containing NaCl (100 mM) and $MgCl_2$ (50 mM) for 1 h at room temperature. After precipitation and removal of the enzyme by centrifugation at 10,000 *g* for 10 min, a sample of the reaction mixture was analysed by LC-MS. This sample was eluted over a C_{18} column using a 15 min gradient of 5-100% acetonitrile and 5 mM ammonium carbonate at a flow rate of 1 mL/min. The mass spectrum was set for positive detection where the product CARP **6** has a m/z = 340.

MIMS assay of catalytic activity for WT ForT and ForT variants.

Membrane inlet mass spectrometry (MIMS) (HDR-20, Hiden)[14] was used to monitor the rate of CO_2 formation in the ForT-catalysed reaction. A solution of 50 mM TES buffer, pH 6.8, containing 4-amino-*1H*-pyrazole-3,5-dicarboxylic acid **5** (1 mM), $MgCl_2$ (2 mM) and WT ForT (6.6 μM) was placed in the reaction chamber (2 mL total volume) and equilibrated for 2 min at 25 °C. Reactions were initiated by the addition of a freshly prepared solution of PRPP **4** (to a final concentration of 2 mM), and CO_2 production was determined in real time by monitoring the ion current at 44 m/z. This procedure was also used to evaluate the Mg-dependence of catalytic activity except that EDTA (10 mM final concentration) was added after 15 min, resulting in the loss of activity. In the case of the ForT variants, which were found to precipitate easily, this procedure was altered so that reaction was initiated by the addition of enzyme (6.6 μM S104A or 46 μM S174A) to the substrates in 50 mM TES buffer, pH 6.8. The ion current signal was converted to the concentration of CO_2 using a standard curve, which was determined by adding known amounts of K_2CO_3 to 10 mM aq. AcOH, pH 2.5. The

CO_2 concentration was calculated using the apparent dissociation constant for H_2CO_3 (pK_a = 6.35) under the assumption that all of the H_2CO_3 dissociates into CO_2.[15]

Molecular docking studies.

The X-ray crystallographic coordinates of the ForT/PRPP complex at 2.5 Å resolution were used to build the initial model of the enzyme for molecular docking studies using the hybrid probes **7** and **8** (Figure 3a, main text). Missing residues (1-10, 172-179, 206-208) and side chains were built using the tools in Maestro 12.2 (Schrodinger, Inc.), and the Chimera software package.[16] The conformational properties of these regions were validated using the Discrete Optimized Protein Energy (DOPE) protocol.[17] All amino acid residues introduced into the X-ray crystal structure were geometry optimized with all other atoms constrained to their crystallographic coordinates. This model, after being solvated in an orthorhombic box of TIP3P water molecules,[18] was then refined using MD simulations (100 ns) using Desmond v6.0. An initial set of models were then constructed by superimposing the atoms in the phosphate and ribose moieties of **7** and **8** onto the cognate atoms in the PRPP molecule bound within the enzyme.

Supplementary References

1. T. Zhou, M. Daugherty, N. V. Grishin, A. L. Osterman, H. Zhang, Structure **2000**, *8*, 1247-1257.
2. R. H. White, Biochemistry **2011**, *50*, 6041-6052.
3. Y.-J. Moon, J. Kwon, S.-H. Yun, H. L. Lim, J. Kim, S. J. Kim, S. G. Kang, J.-H. Lee, S. I. Kim, Y.-H. Chung, Int. J. Mol. Sci. **2015**, *16*, 9167-9195.
4. X. Robert and P. Gouet, Nucl. Acids Res. **2014**, *42*, W320-W324.
5. B. R. Miller, Y. Kung, PLoS ONE **2018**, *13*, e0208419.
6. H. Liu, J. H. Naismith, Prot. Exp. Purif. **2009**, *63*, 102-111.
7. H. Liu, J. H. Naismith, BMC Biotechnol. **2008**, *8*, 91 (doi:10.1186/1472-6750-8-91).
8. G. Winter, D. G. D. G. Waterman, J. M. Parkhurst, A. S. Brewster, R. J. Gildea, M. Gerstel, L. Fuentes-Montero, M. Vollmar, T. Michels-Clark, I. D. Young, N. K. Sauter, G. Evans, J. Appl. Crystallogr. **2010**, *43*, 186-190.
9. G. Winter, Acta Crystallogr., Sect. D **2018**, *74*, 85-97.
10. A. J. McCoy, R. W. Grosse-Kunstleve, P. D. Adams, M. D. Winn, L. C. Storoni, R. J. Read, J. Appl. Crystallogr. **2007**, *40*, 658-674.
11. P. Emsley, K. Cowtan, Acta Crystallogr., Sect. D **2004**, *60*, 2126-2132.
12. M. P. Tarazona, E. Saiz, J. Biochem. Biophys. Methods **2003**, *56*, 95-116.

13. D. Ren, S.-A. Wang, Y. Ko, Y. Geng, Y. Ogasawara, H.-W. Liu, Angew. Chem. Int. Edn. **2019**, *58*, 16512-16516.
14. X. Sheng, W. Zhu, J. Huddleston, D. F. Xiang, F. M. Raushel, N. G. J. Richards, F. Himo, ACS Catal. **2017**, *7*, 4968-4974.
15. M. E. G. Moral, C. K. Tu, N. G. J. Richards, D. N. Silverman, Analyt. Biochem. **2011**, *418*, 73-77.
16. E. F. Pettersen, T. D. Goddard, C. C. Huang, G. S. Couch, D. M. Greenblatt, E. C. Meng, T. E. Ferrin, J. Comput. Chem. **2004**, *25*, 1605–1612.
17. M.-Y. Shen, A. Šali, Prot. Sci. **2006**, *15*, 2507–2524.
18. W. L. Jorgensen, J. Chandraskhar, J. D. Madura, R. W. Impey, M. L. Klein, J. Chem. Phys. **1983**, *79*, 926-935.
